# Comparison of Radiochemical and Chemical Impurities in Liquid Wastes of Two Different ^68^Ge/^68^Ga Generators used in Nuclear Medicine PET Chemistry

**DOI:** 10.4274/mirt.galenos.2020.58569

**Published:** 2021-02-09

**Authors:** Ayşe Uğur, Olga Yaylalı, Doğangün Yüksel

**Affiliations:** 1 Pamukkale University Training and Research Hospital, Clinic of Nuclear Medicine, Denizli, Turkey; 2 Pamukkale University Faculty of Medicine, Department of Nuclear Medicine, Denizli, Turkey

**Keywords:** Gallium-68, 68Ge/68Ga generator, chemical impurity, GMP

## Abstract

**Objectives:**

Germanium-68/gallium-68 (^68^Ge/^68^Ga) generator eluate contains a number of metal cations that can compete with ^68^GaCl_3_, reducing specific radioactivity. The first step in peptide labeling with ^68^GaCl_3_ is to remove ^68^Ge and several other metals with a long half-life. In this purification step, the elution residue that is passed through the cartridge is collected in glass waste bottles. Waste management is included in good production practices, and in particular, the activity of long half-life ^68^Ge (270.95 days) and other toxic metal levels need to be examined. Our objective in this study is to determine the ^68^Ge activity in liquid waste produced by the generation of ^68^Ga and heavy metal concentrations from the generator column materials and to assess whether it can be disposed of as normal waste.

**Methods:**

Liquid wastes produced by passing the ^68^Ge/^68^Ga generator eluate of 2 different identities via PSH+ cartridge have been analyzed with the inductively coupled plasma mass spectrometry device in the advanced technology application and research center of our university.

**Results:**

The average of the ^68^Ge radioactive pollution was estimated to be 0.142 ppm (μg.mL^-1^) in the liquid waste analysis after passing through the PSH+ cartridge in the pre-elution in the GalluGEN brand generator. While there was no tin (Sn) impurity, it was determined that the average zinc (Zn) was 1.95 ppm (μg.mL^-1^) and the average aluminum (Al) impurity was 10.95 ppm (μg.mL^-1^). While no ^68^Ge radioactive pollution was determined in the iThemba LABS brand generator, the average Sn was 0.098 ppm (μg.mL^-1^), average Zn 48.6 ppm (μg.mL^-1^), and average Al impurity 4.135 ppm (μg.mL^-1^).

**Conclusion:**

All ^68^Ge/^68^Ga generators produced have their own certificates. Metallic contamination in the postmarking waste of ^68^Ge/^68^Ga generators can be different. It would be a safe method to keep these wastes in place until they are dumped into the sewage systems, given their half-lives in terms of long half-life radioactive metallic contamination.

## Abstract (tr)

**Amaç:**

Germanyum-68/galyum-68 (^68^Ge/^68^Ga) jeneratör sağım eluatında ^68^GaCl_3_ ile rekabet edebilen, spesifik radyoaktiviteyi azaltan bir dizi metal katyon bulunmaktadır. ^68^Ge/^68^Ga jeneratörlerinde, elüat bileşimindeki metal kontaminasyonu, kolon matrislerine ve sağım yapılan çözücüye bağlı olarak değişir. ^68^GaCl_3_ ile peptid işaretlemede ilk basamak uzun yarı ömürlü ^68^Ge ve birçok metalin uzaklaştırılmasıdır. Bu saflaştırma basamağında kartuştan geçirilen elüsyon artığı cam atık şişelerinde biriktirilmektedir. Atık yönetimi iyi üretim uygulamalarına dahildir ve özellikle atığın uzun yarı ömre sahip ^68^Ge (270,95 gün) aktivitesinin ve diğer toksik metal içeriğin incelenmesi gerekmektedir. Bu çalışmada amacımız ^68^Ga elde edilmesinde Uğur et al. ^68^Ge/^68^Ga Generator used in Nuclear Medicine oluşan sıvı atıkta ^68^Ge aktivitesini ve jeneratör kolon malzemelerinden kaynaklanan ağır metal konsantrasyonlarını belirlemek ve normal atık olarak atılıp atılamayacağını değerlendirmektir.

**Yöntem:**

Merkezimizde iki farklı kimlikteki ^68^Ge/^68^Ga jeneratör eluatlarının PSH+ kartuşundan geçirilmesi ile açığa çıkan sıvı atıklar, üniversitemiz ileri teknoloji uygulama ve araştırma merkezinde bulunan indüktif eşleşmiş plazma-kütle spektrometres cihazıyla analiz ettirilmiştir.

**Bulgular:**

GalluGEN marka jeneratörde ön elüsyonda PSH+ kartuşundan geçtikten sonra sıvı atık analizinde ortalama ^68^Ge radyoaktif kirlilik 0,142 ppm (μg.mL^-1^), olarak tespit edildi. Kalay (Sn) safsızlığı yokken, ortalama çinkonun (Zn) 1.95 ppm (μg.mL^-1^) ve ortalama alüminyum (Al) safsızlığının 10,95 ppm (μg.mL^-1^), olduğu belirlendi. İThemba LABS marka jeneratör atıklarında ^68^Ge radyoaktif kirliliği tespit edilmezken, ortalama Sn 0,098 ppm (μg.mL^-1^), ortalama Zn 48,6 ppm (μg.mL^-1^) ve ortalama Al safsızlık 4,135 ppm (μg.mL^-1^) tespit edildi.

**Sonuç:**

Üretilen tüm ^68^Ge/^68^Ga jeneratörlerinin kendine ait sertifikası bulunmaktadır. ^68^Ge/^68^Ga jeneratörlerin işaretleme sonrası atıklarındaki metalik kontaminasyonlar farklı olabilir. Bu atıkların kanalizasyon sistemlerine verilmeden önce içeriklerindeki uzun yarı ömürlü radyoaktif metalik kontaminasyonlar açısında yarı ömürleri dikkate alınarak bekletilmeleri güvenli bir yöntem olacaktır.

## Introduction

Gallium-68 (^68^Ga) is a significant radionuclide due to its successful clinical application. Currently, ^68^Ga is manufactured and supplied in preclinical and clinical settings using germanium-68 (^68^Ge)/^68^Ga generator systems (1). The interest in ^68^Ga has grown tremendously in recent years as it has become a routinely used radioisotope in clinical positron emission tomography (PET) imaging facilities around the world. The ^68^Ge has a half-life of 270.95 days (2) and can be used as the main nuclide in radionuclide generator system (3). In this radionuclide generator, the ^68^Ge solid binds to an insoluble, inert carrier and forms a secular radioactive balance with ^68^Ga (T_1/2_=68 minute). ^68^Ga can be eluted from the generator using a suitable solvent.

The limit value of^ 68^Ge fraction in a ^68^Ga solution used in the labeling of radiopharmaceuticals is set as 0.001% in the European Pharmacopoeia monograph (4). With the increase in the age of the generator and increase in the number of elutions performed, the ^68^Ge value may increase in addition to the regular activity. Furthermore, metal impurity from the generator may not be just radionuclides. Toxic metals from the column material are also among the impurities that can compete with ^68^Ga in the complexation reaction. Moreover, zinc (Zn) formation occurs with the decay of ^68^Ga. The presence of non-radioactive metals such as tin (Sn), arsenic, nickel, manganese, and aluminum (Al) that are considered metallic impurities in the ^68^Ge/^68^Ga generator eluate are known (5).

**Figure 1:**
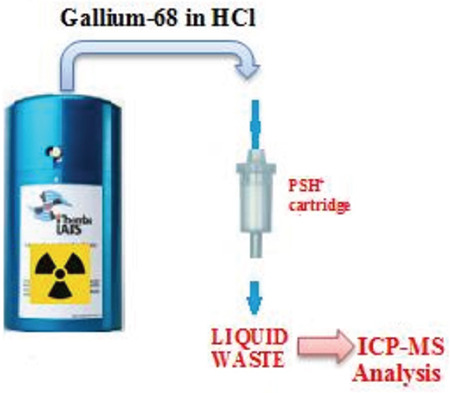
Schematic representation of liquid waste eluting from ^68^Ge/^68^Ga generator ^68^Ge: Germanium-68, ^68^Ga: Gallium-68, ICP: Inductively coupled plasma, MS: Mass spectrometry

Prior to labeling with ^68^Ga in radiopharmaceuticals, the ^68^Ge/^68^Ga generator eluate is subjected to pre-concentration and pre-purification. The various methods used for these processes are based on anion exchange chromatography, cation exchange chromatography, or combination thereof (5,6,7,8). The PSH^+^ cartridge (from cation exchange cartridges) holds pure ^68^Ga; other metals are collected in the waste bottle (Figure 1). We contrasted the two different generator eluates used in our department by separating them from the PSH^+^ cartridge and analyzing the metallic contamination in liquid wastes with inductively coupled plasma-mass spectrometry (ICP-MS).

## Materials and Methods

### Sampling

These 2 generators, which are available at the nuclear medicine department of our university, have different column matrices. The identities of these generators are shown below:

- iThemba LABS (South Africa) ^68^Ge/^68^Ga generator

- PARS Isotope-GalluGEN (Iran) ^68^Ge/^68^Ga generator

GaCl_3_ eluates were obtained from iThemba and PARS Isotope-GalluGEN commercial ^68^Ga/^68^Ge generators with HCl solution in the Scintomics GmbH GRP module 4V synthesis module. In addition, hydrochloric acids (0.6 M ultra-pure HCl, 0.1 M ultra-pure HCl) was obtained from ABX D-01454 Radeberg (Germany). Cation exchange cartridge (PSH^+^, non-preconditioned) (ABX D-01454 Radeberg, Germany) was used to remove metals in GaCl_3_ solution eluated from the ^68^Ge/^68^Ga generator. GaCl_3_ was eluated from the PSH^+^ cartridge with 5.0 M NaCl (ABX D-01454 Radeberg, Germany). Then, 7 mL (n=3) of waste solution was taken to the glass vial for analysis.

### Measurements

The eluate from generators and leftover after ion exchange prior to radiolabeling is acidic and contains a certain amount of ^68^Ge activity (7). Before the analysis, the eluates waited ten half-lives in the vials in compliance with the TAEA transport regulation.

Further, qualitative and quantitative analyses of metal contents in liquid waste were measured at the ppm level using the ICP-MS device located in the advanced technology application and research center of our university.

ICP-MS standard solutions were obtained from PerkinElmer (UK). Moreover, certified levels of standard solutions are as follows: Zn, 998 µg.mL^-1^±5 µg/mL; Sn, 1.002 µg.mL^-1^±5 µg mL; Al, 1.002 µg.mL^-1^±5 µg mL; and Ge, 999 µg.mL^-1^±5 µg/mL. Zn, Sn, Al, and Ge were used as internal standards for ICP-MS analysis. Further, no statistical method was used in the results, and the average of the sample analysis repeats was taken.

## Results

As specified in the generator usage protocols, the generators were regularly eluted every day to avoid high ^68^Ge excretion in the eluate. In both generators, the elutions, generated at one-day intervals, were passed through the PSH^+^ cartridge, and samples (n=3) of the liquids discharged to waste were collected. Each sample (total of six samples) was analyzed three times with ICP-MS, and the averages are shown in Table 1. The average of ^68^Ge radioactive pollution was estimated to be 0.142±0.05 ppm (µg.mL^-1^) in the liquid waste analysis after passing through the PSH^+^ cartridge in the pre-elution in the GalluGEN brand generator. While there was no Sn impurity, it was determined that the average Zn was 1.95±0.05 ppm (µg.mL^-1^) and the average Al impurity was 10.95±0.05 ppm (µg.mL^-1^) ppm. In the iThemba LABS brand generator waste, no ^68^Ge radioactive pollution was calculated; on the other hand, the average Sn was 0.098±0.05 ppm (µg.mL^-1^), average Zn 48.6±0.05 ppm (µg.mL^-1^), and average Al impurity 4.135±0.05 ppm (µg.mL^-1^).

## Discussion

In nuclear medicine PET chemistry, liquid waste is the result of the production of radiopharmaceuticals and is able to contain heavy metals, chemicals, and radioactive compounds (8). Wastes from generator elution used in the production of radiopharmaceuticals with ^68^Ga chemistry in many production centers are left to the sewer. Studies are performed on the reduction of ^68^Ge activity in liquid waste and disposal of radioactively contaminated waste in nuclear medicine ^68^Ga PET chemistry using a recirculation system with a sorbent (9,10). The ^68^Ga radionuclide used in PET chemistry is typically obtained using commercial SnO_2_- or TiO_2_-based ^68^Ge/^68^Ga generators. It has been reported that the cleaning level of ^68^Ge activity in wastes cannot exceed 10 Bq/g in the European Directive 96/29/EURATOM (11). The amount of ^68^Ge in the elution specified in the generator manufacturer’s certificates is ^68^Ge <0.001% of nominal activity. The exemption concentrations and exemption activities of radionuclides in IAEA Safety Standards are shown in Table 2. The exemption limit for ^68^Ge is 1x10^1^ (Bq/g) (12). Column materials are specially filled and approved for each of the ^68^Ge/^68^Ga generators used in clinical pet chemistry. Radioisotopes with a half-life of more than 100 days are not covered by the TAEA regulation; it is understood that they must be delivered to the National Storage Centers when they have exhausted their useful lives. The recycling or subsequent use of this radioisotope outside the specified landfill or reintroduction into the economic cycle should be strictly excluded. After passing through the PSH^+^ cartridge of the PARS Isotope-GalluGEN brand (10-month) generator from 2 different generators that we used in our study, we determined the ^68^Ge radioactive pollution in the liquid waste above the value of 0.000036% specified in the certificate.

At the same time, the toxic metal threshold concentrations in liquid wastes were determined by the Hazardous Waste Control Regulation. ^68^Ga decays with a half-life of 68 minutes to stable ^68^Zn. After the iThemba brand generator elution, the Zn impurity in the waste is estimated to be 48.6 ppm, well above the 10 ppm value specified in the certificate. Waste resulting from the production and preparation of pharmaceutical products included in the “hazardous waste category according to their natural character or the activity that creates them” of the Hazardous Waste Control Regulation are evaluated in compliance with Annex 5 of the same regulation (13). According to the regulation, a highly toxic substance has a total concentration ≥0.1%, toxic substance at total concentration ≥3%, and harmful substance at total concentration ≥25%. In our study, Zn, Al, and Sn determined at the ppm level are below the 0.1% level defined in the regulation and toxic metal class. Zn pollution in waste is above the 10 ppm limit value specified in the certificate of the generator; it is also below the maximum toxic metal limit for the recycling of waste.

## Conclusion

In our study, the toxic metal contents determined at the ppm level for both generators are below the levels to be specified in international regulations. In addition, increased metallic impurities associated with the increasing age of generators are an expected result. For aged ^68^Ge/^68^Ga generators, it is recommended that the generators pass the milking products through the PSH^+^ cartridge and hold for long half-life radioactive metals (especially for ^68^Ge) before they are released into the sewer.

### Disclosure Statement

The author has no personal interest in the commercial suppliers of ^68^Ge^/68^Ga generators or ^68^Ga-labeled imaging pharmaceuticals.

## Figures and Tables

**Table 1:**
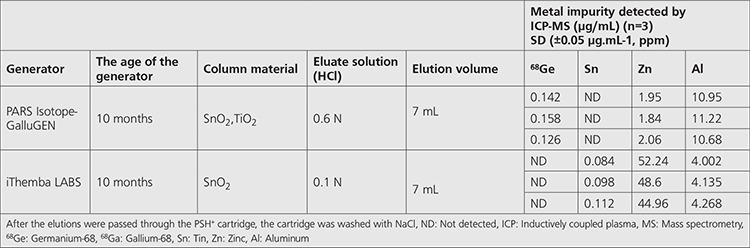
Comparison of the metal contents in the elution after passing the 2 different ^68^Ge/^68^Ga generator elutions through the PSH^+^ cartridge (n=6). Elution conditions

**Table 2:**
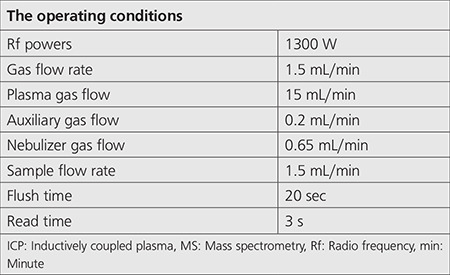
The operating conditions of the ICP-MS device
